# Expanding the green gaming horizon: a conceptual analysis of and proposed guidelines for upscaling environmental game usage in climate education

**DOI:** 10.3389/fpsyg.2024.1414705

**Published:** 2025-01-21

**Authors:** Kristoffer Skomsøy Fjællingsdal

**Affiliations:** Department of Psychology, Norwegian University of Science and Technology, Trondheim, Norway

**Keywords:** environmental games, serious games, upscaling, sustainability, science communication

## Abstract

Despite the now unequivocal notion that climate change is driven by anthropogenic activity, communication between concerned climate scientists and laypeople about the severity of the issue is still muddy. Although creative and more approachable venues of communication to climate change and sustainability issues are being explored more regularly than before, there is still room for improvement and upscaling in the attempts to link scientists and laypeople together in the understanding of these outstanding issues. This also applies to the field of environmental gaming, which has become more popular in the recent decade. Despite this increasing popularity, however, most environmental gaming studies exist as small-scale pilot studies that often result in generating limited, albeit promising results in terms of increasing awareness and knowledge around environmental topics. This article explores the use of games in climate- and sustainability education and provides a set of assisting guidelines to ease the process of using games as communication tools about these pressing issues, as well as providing advice on how to upscale environmental gaming from a set of limited pilot studies.

## 1 Introduction

Climate change, biodiversity decline, the energy crisis—these represent just a handful of the wicked problems that the world is facing today (IPBES, 2019; IPCC, 2023), and the science showcasing the role of anthropogenic activity in this is unequivocal (Somerville and Hassol, 2011). Paradoxically, it is also well-known that science communicators and scholars alike are struggling to engage meaningfully with laypeople in terms of showcasing exactly how dire these wicked problems truly are (e.g., McBean and Hengeveld, 2000; Moser, 2016; Wang and Coren, 2024, p. 5) and why they need public action to be circumvented. As a result, the need for creative approaches to environmental communication is growing (e.g., Illingworth, 2020; Redfern et al., 2016). One such approach comes in the form of *environmental games* (sometimes referred to as green games, climate games or sustainability games), which have the explicit purpose of teaching, communicating or otherwise informing their players of general as well as specific issues that fall under the umbrella of anthropogenic climate change.

In 2015, embarking on the road toward investigating the transformative potentials in environmental games as part of his PhD (Fjællingsdal, 2021), the author sent an email to Jesse Schell, game designer and author of the book “The Art of Game Design: A Book of Lenses”, asking his opinion as to why games about the climate and the environment are less attractive or popular than the more conventional or commercially successful games that were available on store shelves. This question is important due to two main reasons; firstly, due to the current state the environmental gaming market, which had bloomed significantly in the years prior (Reckien and Eisenack, 2013) despite failing to enter mainstream gaming, and secondly, the steadily increasing popularity of gaming (ESA, 2015)—a rise that continues to this day (ESA, 2023). His response was quick and to the point: (1) many environmental games are of inadequate quality, and (2) they are harder to make due to having more constraints than conventional games—or, in his words: “Making a delicious pastry is difficult; making a delicious pastry that cures cancer is much harder” (J. Schell, personal communication, September 11, 2015). It is now 9 years later, and a recent article shows that despite developments in the serious gaming field, we are still left 'paddling in the shallows' in terms of tapping into the deeper potential in games for educational or transformational purposes (Heron and Crabb, 2023). This also holds true for the gradually emerging field of environmental games, although they have been subjects of research since at least the 1980s (Baba et al., 1984; Robinson and Ausubel, 1983).

There appears to be a notion among game scholars and -researchers that games both can and should be utilized in educational contexts such as classroom activities and curricula or other organized initiatives—a notion that goes back at least several decades (e.g., Abt, 1970). Despite this, as well as the increasing fervor, activity, and contributions that can be observed among the gaming field's many fandoms (Heron and Crabb, 2023), meta-analyses across a span of several years on the general effectiveness of games as educational tools tend to yield mixed or inconclusive results (e.g., Arztmann et al., 2023; Girard et al., 2012; Talan et al., 2020) even though they are often shown to be more motivating, engaging, accessible and fun to use than other forms of educational tools (Gee, 2005; Jennett et al., 2008; Ryan et al., 2006). For environmental games specifically, few such analyses appear to even exist—something which can largely be attributed to an overall lack of empirical research on them (e.g., Hallinger et al., 2020). Although these analyses on the effectiveness of environmental games have generally yielded promising results (Boncu et al., 2022; Janakiraman et al., 2021a,b; Rajanen and Rajanen, 2019), they still have significant barriers working against their use (Fernández Galeote et al., 2021) and upscaling—that is, their potential for being used for purposes other than lone-standing pilot studies. Heavily inspired by the design and structure of Gifford's (2011) seminal work on psychological barriers against pro-environmental behavior, this paper seeks to address barriers against the use of games in climate- and sustainability education, with the goal being to create a set of proposed guidelines that can illuminate how games can be used to maximize their effectiveness as communication tools about climate change and its underlying facets. These guidelines are intended to help future researchers and practitioners (e.g., teachers, pedagogues, and environmental gaming enthusiasts and -hobbyists) who wish to use environmental games in their projects, provide a realistic picture of how complex yet gratifying gaming interventions can be, as well as underline what can be considered necessities to upscale environmental gaming research beyond limited pilot studies—an issue that has proven to be pervasive across the environmental gaming research literature (Fernández Galeote et al., 2021).

## 2 Environmental games—What are they, and how can they be used?

As previously stated, an *environmental game* can be defined as a subgenre of *serious game*—a game that seeks to do more than just entertain its players (Abt, 1970). Environmental games seek to educate, inform, or otherwise communicate about general or specific issues relating to climate change and other topics within the frame of sustainability, as well as build awareness and motivate people to act against climate change (Ouariachi et al., 2018, p. 1). They come in both digital (computer-, console- or browser-based games) and analog formats (board- and card-based games), as well as varying degrees of complexity. Early examples of digital environmental games include the environmental policy game *Balance of the Planet* (Crawford, 1990), and the planetary development game *SimEarth* (Wright, 1990)—both containing elements that can be found in newer digital environmental games such as *Fate of the World* (Red Redemption, 2011). Social dilemmas, some of which constitute the root of the environmental issues we are facing (Dawes, 1980), are also subjects of environmental games. One example includes the *Commons* game (Powers, 1987) which illustrates how Garrett Hardin's *Tragedy of the Commons* operates—a theoretical situation in which different actors are provided a shared resource and need to co-manage it (Hardin, 1968). Another example—also revolving around the core tenets of Hardin's Tragedy of the Commons—is the hybrid digital-/board game *Fish Banks* (Meadows, 1986), where the players must operate their own competing fishing companies while simultaneously trying to prevent the fish stocks from depleting entirely. Some environmental games are simplistic, revolving around basic actions such as reducing energy use in the home (Klöckner, 2015, p. 199), while others are developing into large-scale, complex climate simulators in close collaboration between climate scientists, professional game designers, and the players themselves (e.g., *Eco*, by Strange Loop Games or the Corporation for Public Broadcasting-funded *World Without Oil* project). Some environmental games follow a classic instrumental design approach where they provide didactic content to the player about how to behave sustainably, whereas others utilize a humanistic design approach where the players are given more agency to determine what constitutes environmental issues and how to counteract them (Spanellis et al., 2024). Furthermore, some environmental games are commercially available off-the-shelf (e.g., *Keep Cool* by Eisenack and Petschel-Held, 2004 and Fate of the World) whereas others exist solely as temporary pilots for scientific studies and are not available to the general population.

Despite the overall lack of large-scale empirical research on the use of environmental games, they are generally shown to be capable of breaking down some of the psychological barriers surrounding pro-environmental behavior as described in Gifford (2011). These include (a) emphasizing climate change as a local and personal risk, (b) encouraging more affective and experimental solutions to the issue, (c) appealing to relevant social groups, (d) emphasizing which policies can lead to immediate action, and (e) focusing on environmental goals and outcomes that hold long-term value (van der Linden et al., 2015). Environmental games are shown to reduce the psychological distance to environmental issues and making them appear closer and more relevant to the individual by holding the player accountable for their impact on the in-game world (Fjællingsdal and Klöckner, 2019). In many ways, sophisticated environmental games can be considered *microworlds—*simplified representations of reality containing artifacts that the player can freely explore, experiment with, and understand (Egenfeldt-Nielsen, 2006). Through learning in this manner, players are allowed to make mistakes and explore the effects of their own manipulations and impact, without having to fear real-life consequences (McGonigal, 2011, p. 303). Some games, such as the board-based *Evolution: Climate* (Crapuchettes, 2016), also utilize affective components that can generate empathy with animals or other living things that can be negatively affected by a drastically warmer or colder climate. Others allow their players to experiment with various environmental policies to see how it affects the state of the in-game planet, such as Fate of the World (Red Redemption, 2011). Lastly, some games allow the expression of alternate worldviews, political and religious affiliations, and motivations by situating the players in shared social dilemmas (Fjællingsdal and Klöckner, 2019, 2020; Flood et al., 2018; Wu and Lee, 2015).

## 3 Methods

To explore the concept of environmental games and generate proposed guidelines for their upscaling, a conceptual analysis was conducted (inspired by Ho and Sommers, 2013). Although the chosen conceptual analysis framework consists of 8 steps, 1 of them (identification of antecedents and outcomes) was omitted from this paper due to being more relevant for clinical or medical fields of study. The chosen conceptual analysis framework therefore consists of 7 steps: (1) concept selection, (2) analysis purpose, (3) concept use identification, (4) identifying defining attributes, (5) identifying empirical referents, (6) identifying model cases, and (7) identifying borderline, related or contrary cases. A preliminary literature search for the key terms “environmental games”, “sustainability games” and individual game names (e.g., “Eco”) was conducted in Google Scholar to provide insight into the concept of environmental games, establish a clear purpose for analyzing them in light of identifying barriers toward their upscaling, and gain an understanding of the defining attributes of environmental games and how they are used in empirical research (Steps 1–5). Additionally, a parallel search for environmental games on the gaming aggregates Steam and BoardGameGeek was also performed to identify representative environmental games for a conceptual analysis (Steps 6–7). The literature search revealed 52 games with an environmental focus, and that the number of new environmental games grew considerably since the early 2000s (Reckien and Eisenack, 2013). This trend also appears to extend to the quality, rigor, and frequency of scientific research on the topic (Fernández Galeote et al., 2021; Hallinger et al., 2020). The literature review also showed that environmental games remain a niche form of serious game with few empirical applications (Hallinger et al., 2020), and even fewer interventions on a larger scale than pilot studies (Fernández Galeote et al., 2021)—one notable exception being the World Without Oil project where individuals across the world contributed ideas on how to solve a fictional global oil shortage crisis (McGonigal, 2011). Recurring games discovered in the literature search (e.g., Eco and Keep Cool) were then cross-checked with their Steam and BoardGameGeek pages where the games were commercially available, and their overall user ratings were used as a basis for inclusion in this conceptual analysis. Based on the literature and games explored, a set of proposed guidelines has been generated and will be explored further in the following section.

## 4 Using and upscaling environmental games—A proposed set of guidelines

In the same vein as other forms of serious games, environmental games have a range of issues and barriers surrounding their implementation by academics and practitioners wishing to tap into their inherent potential as communication and learning tools. Several of these issues and barriers can be directly related to the overall lack of upscaled gaming interventions that are sorely needed to advance the field today. The following section is dedicated to illuminating and exploring some of these issues and barriers, as well as relating them to the overarching issue of scalability. Although this list is not likely to be entirely exhaustive, it does serve as an important early step toward the upscaling of environmental games as a creative, engaging, and approachable way to learn about climate and sustainability topics.

### 4.1 Environmental games and the social component—By humans, for humans

The social component of game design and -use is inevitable. As Schell (2010) shows, each step of both game design and gameplay involves deep consideration and reflection from the designers themselves and what ideas or visions they have for how the game is supposed to look, as well as how the players eventually receive, decode and appreciate the designers' vision. Any successful game-based learning intervention therefore starts with good preparation and surveying which games are available on the market, as well as what they involve in terms of their contents and playtime, complexity, player-, software- and hardware requirements, and relevance to the subject that is to be taught. Failing to conduct a solid survey of these factors could result in a suboptimal gameplay experience, a missing link between what the game can teach and the overall aim of the game-based learning intervention, or even a possibility that the game is unable to be played at all due to limitations in the software or hardware that the games require. As such, when choosing a suitable green game for a given intervention, it is vital to understand the relevance and complexity of the game's contents in relation to its players as well as its suitability for the intervention's framework in terms of gameplay location, time use, and other contextually relevant factors. Although there is an overreliance on the inherent, almost magical properties of a game to teach about its subject matter (Gunter et al., 2007), a considerable amount of work is involved in preparing and conducting the intervention itself. As it stands, there is a likelihood that pilot studies that underestimate or otherwise fail to acknowledge the preparatory stages of gaming interventions are likely to yield disappointing research findings, as well as resulting in a lack of continued interest in game-based learning or expanding the gaming intervention past its pilot stage. This section is therefore intended to address known issues and pitfalls in the use of game-based interventions, to improve the outcomes and increase the likelihood of moving these interventions from pilots into upscaled environmental gaming research projects.

#### 4.1.1 Teamwork is key

Gaming interventions often involve significant preparatory work—the field needs to be surveyed to find a game that is appropriate for the intervention, gaming licenses or physical games must be acquired, rules must be examined and understood, software and hardware checks must be conducted, physical spaces and other necessary requirements need to be booked in order for the intervention to take place, a research design needs to be decided upon if research on the intervention is intended, players must be recruited, expectations and goals for the game must be clarified, and significant gameplay dates need to be set. Already, the list of preparatory work might appear daunting—but it is hardly exhaustive. Individual environmental games have individual requirements, and some of them have been shown to take a significant amount of time to complete (Fjællingsdal and Klöckner, 2019) or even explain to the players (Fjællingsdal and Klöckner, 2020). As such, it is recommended that gaming interventions are planned and organized by dedicated groups with relevant expertise, and preferably featuring instructors or facilitators who are familiar with the game and its mechanics from before (Flood et al., 2018). Early engagement with a team of relevant actors (e.g., teachers and the IT department at a school) (Skaug et al., 2020, p. 161) or local volunteer gaming centers or hobby groups can ease the planning process of gaming interventions, as well as providing potential insight into expectations and preconceptions surrounding the intervention itself. It is also recommended that the team conducts some gameplay sessions beforehand—both to get acquainted with how the game plays and works, but also to gain inspiration as to how the game can be used to maximize its learning potential.

#### 4.1.2 Using games co-developed by professional game designers and climate experts

While the gaming market is seeing an influx of new games with environmental themes (Hallinger et al., 2020; Reckien and Eisenack, 2013), only a select few of them are developed as collaborations between professional game designers and trained academics. Fewer still are those games who also end up receiving good or decent reviews on some of the most widely used online gaming platforms such as Steam and Metacritic. Examples of these select few include digital games such as Fate of the World (Red Redemption, 2011) and Eco (Strange Loop Games, 2020), and board games such as Keep Cool (Eisenack and Petschel-Held, 2004). As mentioned earlier in this article, Jesse Schell stated that the main reasons for why environmental games might fail boil down to (1) their overall lack of quality, and (2) having more constraints due to their more “serious” nature. Although several academics tend to develop their own games for the purpose of educating or generating awareness of certain issues, they often lack the creative rigor and *gameful* experiences that only a career in game design can hope to enable (Theodosiou and Karasavvidis, 2015)—*gamefulness* referring to the psychological state in which one playfully or joyfully approaches in-game goals and tasks as non-trivial obstacles that need to be overcome (Landers et al., 2019). As such, it is recommended that games co-developed by professional game designers and academic scholars should be utilized more. Dedicated researchers wishing to use such games are encouraged to survey the field thoroughly, using the tagging systems on larger gaming aggregates such as Steam or BoardGameGeek to locate games with environmental themes, reading reviews of relevant games on Metacritic to gain insight both from professional and public reviewers, and engaging with forums and fandoms dedicated to the game in question.

#### 4.1.3 The target audience

Most games have a target audience—the type of player that the game is intended for. Although game designers tailor their games for selected people (Schell, 2010, p. 97), formal designation of the target audience by rating boards such as the Entertainment Software Rating Board (ESRB) is also required, which normally uses demographic factors (e.g., age) and the level of the game's mature content (e.g., violence or language use) as a basis (ESRB, 2024). These rating scales, however, generally concern the suitability of the game's content, not complexity, for the players based on their psychological maturity. More recently, websites such as BoardGameGeek.com has begun introducing complexity scales (otherwise known as *weight*) which detail factors such as the difficulty of the game's rulebook, how long the gameplay time is, the amount of time spent planning one's actions in the game, whether the game is luck- or skill-based, the amount of technical skill required to play, and how many replays are required in order to fully understand the complexities of the game (BoardGameGeek.com, 2024). For digital games, websites such as HowLongToBeat.com provide an overview of how long previous players of the game, on average, have taken to beat specific games given specific scenarios, which makes it easier to choose an appropriate game for timed interventions particularly. Using such guides before a gaming intervention simplifies the process of choosing appropriate games, even though they are seldomly operationalized—seemingly relying more on player feedback than scientific evaluation. It has been shown, however, that the often-daunting complexity of games can be alienating (McNamara et al., 2009), thus highlighting the need for such scales. As an example, Eco—although being met with critical acclaim—has been shown to be quite labor-intensive, sometimes requiring up to 30 real-life days of gameplay to meet the in-game goal (Fjællingsdal and Klöckner, 2019). On the opposite end of the scale, other environmental games are very simplistic and aimed for younger audiences, meaning they take little time to complete and feature little complexity (Koehler et al., 2017). For a dedicated researcher wishing to do gaming interventions, understanding the connection between game complexity, game content and the target audience is therefore important. Failure to use an appropriate game for a specific audience could, in many cases, lead to frustration, boredom, and disappointing research findings. This is concurrent with the notion that intrinsic motivation to act arises from an ideal relationship between the complexity of a task and the skill level of the person performing said task—the so-called *zone of optimal experience* (Csikszentmihalyi, 1990).

#### 4.1.4 Game skepticism

Although environmental games are not a new concept, the small-scale empirical evidence on their effectiveness (e.g., Hallinger et al., 2020) as well as varying degrees of complexity, unfamiliarity and often unclear connection to mandatory school curricula (Klopfer et al., 2009) have exerted a certain skepticism among teachers, students, and parents alike in terms of using them—especially in classroom settings or pilot studies where serious games are generally meant to be played. In a classroom setting, for example, games have been frowned upon for their lack of graphical quality (Rice, 2006), confusing or complex user interfaces (Fjællingsdal and Klöckner, 2017), low production values and insufficient gameplay quality (Illingworth and Wake, 2019). Some educational games are also scrutinized for being unrealistic or failing to depict the full complexity of environmental issues, although past research has shown that players are often capable of discounting the game's lack of realism (Feinstein and Cannon, 2002; Norman et al., 2012) as well as asking critical questions as to why some things work in the game but not in real life (Schell, 2010). This especially appears to be the case for more experienced players who are familiar with realistic breaches in games (Fjællingsdal and Klöckner, 2019). However, much of the criticism is still warranted due to the aftermath of the influx of low-quality e-learning and digital education tools and -games that began flooding the market in the early 1990s. Much has since changed for the better—also in the environmental gaming field (Reckien and Eisenack, 2013) but dispelling the prevalent skepticism toward games is still a complicated matter. Game skepticism furthermore becomes a negative spiral in that it discourages their use, which in turn ensures that empirical investigations into their effectiveness will remain low. One possible solution to this is to engage with the relevant stakeholders (e.g., teachers, students, and parents if the game is to be used in a classroom setting) at an early stage in the gaming intervention—weighing the interest toward using games and acknowledging any barriers toward using them. Research has shown that allowing skeptical teachers to engage meaningfully with an educational game is both capable of (1) dispelling some of the existing skepticism, as well as (2) allowing for an open-ended discussion where any remaining concerns surrounding the use of games in a specific setting can be addressed and clarified (Gaudelli and Taylor, 2011). This research has also shown that teachers are generally cautious of using educational games because of the way in which they present and trivialize their content, take too much time to plan and implement, and that they feel that games can never “outperform them” as teachers. Acknowledging such insecurities and finding solutions for them at an early stage of an environmental gaming intervention is therefore recommended to ease the adoption process and fitting the chosen game into the curriculum. One way in which to do this is through the organization of short workshops before the main intervention itself, where skeptical stakeholders can engage with the game and voice any remaining concerns they might have.

### 4.2 Learning objectives and openness to new horizons

Another core issue with environmental games that prevents their upscaling is the relative newness of their application as educational tools, despite having been formally included in research since at least the 1980s. Combined with the fact that most educational institutions have a clear demand that all of their institutional activities must in some way, shape or form be connected to the mandatory school curriculum (Skaug et al., 2020, p. 73), implementation of games in educational settings can be challenging. This can also lead to games being used as convenient and colorful wrapping for otherwise boring or didactic content (Galarneau, 2005), meaning that many educational games (and not just environmental ones) stand the risk of simply becoming digitized textbooks rather than the “magical” education tools that serious gaming literature often paints them as. Although this approach might be decent, as Galarneau observes, for learning material that is otherwise mundane and boring or requiring repeated practice to fully comprehend, it is not likely to be a very engaging means of communicating about dramatic and complex issues such as climate change or biodiversity loss.

#### 4.2.1 “Beyond” the curriculum

As previously stated, games need to fit the curriculum if they are to be utilized in conventional educational settings such as schools. It is worth noting that in some of these institutions, although the curriculum contains clearly stated learning objectives, there is still a significant amount of wiggle room in terms of using games as cultural expressions within the classroom setting (Skaug et al., 2020, p. 73). And while the notion today is that games need to adhere vehemently to the curriculum, it is possible to argue that they can go well beyond it too. Imagine, for example, a situation in which a biology curriculum states that the learners need to obtain a basic understanding of how different species relate to each other in an ecosystem. The relationship between different species in a frail ecosystem is a core component of the digital game Eco (Möring and Schneider, 2024, p. 209), which has been mentioned earlier in this paper. But the game also situates this theme within a much larger context—for example, the relationship between basic physiological human needs and animal products, the need to gather materials necessary for the survival of other species in order to build necessary infrastructure, the effect of anthropogenic emissions on nature, as well as the overarching and highly important (albeit fantastical) scenario that an asteroid is set to hit and severely damage the in-game planet in 30 real-life days (Fjællingsdal and Klöckner, 2019). The game therefore does not statically communicate the complexity of the relationship between species with text; it rather gives the player agency in what happens and why, as well as how it impacts the game world—thus generating a form of *designed experience* where emotional pathways are formed between the in-game action and the in-game consequence of that action (Wu and Lee, 2015). This form of imparting knowledge (or persuasion) is known as *procedural rhetoric*—a practice of using processes and rules rather than the more conventional and static text and images; altogether, a promising communication approach showcasing how things work or are connected (Bogost, 2008, p. 125). Games also do not necessarily need to be explicitly educational to form interesting discussion backdrops about environmental topics; even conventional, highly lauded, and off-the-shelf games have tackled the topic before (Blake et al., 2024). One is the 1997 PlayStation game *Final Fantasy 7* (Square, 1997) where the player is set to fight against a large global energy company which threatens to annihilate the planet by drawing out its life energy. Although this is not the only goal in the game, it represents a significant part of the background for its main storyline, and parallels can certainly be drawn to the ongoing real-life energy crisis. Another example is the digital game *Okami* (Capcom, 2007), where some of the mechanics in the game revolve around restoring twisted, damaged nature with magical brushstrokes. Environmental themes can also be found in the digital game *Oddworld: Stranger's Wrath* (Oddworld Inhabitants, 2005), where the player is set to combat an entity responsible for species extinction and monopolization of natural resources. Although these games are only tangentially environmental, they can still serve as a more approachable introduction or backdrop to contemporary climate issues than a conventional textbook and could potentially represent an answer to the call for more engaging means of pro-environmental communication (Illingworth, 2020). It has also been found that, while explicitly educational environmental games might be less capable of reaching wider audiences, conventional off-the-shelf games often contain educational properties about nature elements such as wildlife species (Crowley et al., 2021)—meaning that they can form a solid foundation for discussion and reflection when used by a creative educator or practitioner.

#### 4.2.2 Knowledge is power—But not action

Knowledge about environmental issues is an important, yet insufficient component of pro-environmental behavior (Sturgis and Allum, 2004). This is crucial in an upscaling perspective, as environmental games are primarily intended to motivate climate action (Ouariachi et al., 2018, p. 1). Yet—again paradoxically—the primary goal of environmental games often tends to be knowledge provision in some form. As a result, as Galarneau (2005) has previously observed, they are often perceived as digital copies of conventional textbooks—that is, not very engaging and not necessarily a good alternative to traditional teaching methods. It can be argued that a shift in the perspective of environmental games usage, or perhaps serious games usage in general, from a strong focus on knowledge provision to more innovative applications is warranted. As Bogost (2008, p. 125) notes, games do not primarily teach by having their players read static text and images—they teach and immerse their players by *having them do things in a context dictated by in-game rules*—solving puzzles, fulfilling quests, becoming stronger, visualizing their in-game impacts, and understanding complex correlations between their actions and consequences. Knowledge and information in this context do not become secondary to the gameplay experience, as some have been shown to be skeptical of (see Section 4.1.4—Game skepticism); they are merely presented and viewed in different formats. One important note in this regard for the future of green games is to elevate their standing from simple knowledge-provision tools to socially constructed, holistic experiences—a microworld or open-ended universe in which a topic is represented by different artifacts that the player can interact with on their own leisure (Egenfeldt-Nielsen, 2006) and through which they can gain a deeper understanding of the topic from their own referential standpoint, context, and life situation. Also, when combined with a debriefing session (see Section 4.2.3—The importance of debriefing), environmental games can become highly effective educational tools (Madani et al., 2017).

#### 4.2.3 The importance of debriefing

Perhaps the most important part of playing environmental games (aside from the quality and thematic relevance of the games themselves) is the *debriefing* session. The field of educational games in general is characterized by a multitude of learning myths, including notions such as “serious games requiring no debriefing” (Crookall, 2023), which can ultimately be detrimental to the game's educational value and continued use. During a debriefing, participants are normally asked to reflect upon their experiences with the game, how it made them think and feel during the action, whether they learned anything new or reinforced their existing knowledge somehow, as well as core aspects of the more experiential and phenomenological aspects of the gameplay session (Lederman, 1992). It also represents an arena through which an instructor can pose challenging and thought-provoking questions regarding the game experience, address existing and missing parallels between the game and the real world, and to justify or discuss the inclusion of fantastical rather than realistic elements for game enjoyment purposes. As such, it is therefore an important component in bridging the *simulation gap—*a discrepancy between the actions performed by a player in a serious game compared to a real-life setting (Bogost, 2010, p. 43)—by encouraging, stimulating, and enabling pro-environmental behavior to be conducted outside the game itself. One core misconception about debriefing, however, is that it is exclusively meant to take place *after* the game has been played—or, as one article headlines it, “the real learning begins when the game *stops*” (Tipton et al., 2016). It can be argued that some aspects of debriefing can begin *during* gameplay through casual observation of- and interaction with the players—focusing on gestures or the strategies the players implement in-game for example (Crookall, 2023). In so doing, educators and researchers can perceive and make note of behavioral and affective patterns that would otherwise be inaccessible in a post-game debriefing. Debriefing from educational gameplay today is often remarkably shallow and only covers the pure basics of a gaming experience, thus leaving the players with only the experiential aspects of the game and possibly not seeing the extended educational value of what they have played (Tipton et al., 2016). When done correctly, debriefing can be used to ask questions or provide feedback throughout the gaming session before culminating in a reflective process once the gameplay session ends. Using a more inductive, bottom-up approach to questioning, debriefing can also encourage the players to openly talk about their gameplay experience to catch unknown or unpredicted aspects of what the game is capable of teaching (e.g., Fjællingsdal and Klöckner, 2019, 2020). Additionally, although debriefing traditionally occurs face-to-face once the gameplay session is concluded, online instant messaging tools such as Discord or Google Forms can both (1) enable easier and more direct communication during the gameplay session, as well as (2) provide the players with the opportunity to participate in individual- or group-based online interviews about their experiences in the game (Crookall, 2023).

### 4.3 Upscaling environmental game usage

Games have been shown to be able to teach and inspire us about various themes and subjects (Boncu et al., 2022; Janakiraman et al., 2021a,b), but it can be argued that their true potential in encouraging sustainable behavior through a wider application than pilot studies remains untapped. In the previous part of this paper, a set of important factors to upscaling environmental games has been presented. These factors are also of utmost importance to utilizing the educational potential of these types of games, but they are often given little attention in contemporary research. As such, they form a potential scaffolding for the expansion and upscaling of pilot-level gaming studies. They furthermore address some of the most common barriers and hurdles toward implementing environmental games as educational tools. Currently, there appears to exist a hesitation to use games extensively to teach about the climate and the environment. Yet, despite this, there is an outcry for new and innovative means of engaging with new learners and investigating alternative arenas through which the complexity of the ongoing social crises can be explored (e.g., Illingworth, 2020; Redfern et al., 2016). Breaking down these barriers and skepticism is crucial to ensure the increased uptake of games in environmental education, and the previous sections of this paper are intended to help achieve this goal.

## 5 Conclusion

The purpose of this paper has been to provide a basic set of guidelines surrounding the use of environmental games across various fields of climate education, and to avoid common pitfalls and barriers that could ultimately prevent their upscaling from small-scale pilot studies. To this end, a conceptual analysis of the environmental gaming genre was conducted based on a combination of a literature search and an exploration of popular gaming aggregates. The analysis has revealed that despite the increasing numbers of environmental games on the market—and the frequency of research into their effectiveness—there still exist significant barriers to their use and upscaling potential that this paper will hopefully help ameliorate, and although the list is likely not entirely exhaustive it will still be a helpful tool for those who wish to use environmental games in their educational activities. It should also be noted that this paper does not suggest replacing conventional learning methods with environmental games; rather, it is suggested to consider that these educational tools are complementary. Lastly, future empirical evaluation and possible expansion of the guidelines described in this paper is encouraged.
